# An Exploratory Study on Two Exogenous Estrogenic Metabolites (EDMs): Their Effects In Vitro and on Oophorectomized Female Rats

**DOI:** 10.3390/metabo16090647

**Published:** 2026-09-03

**Authors:** Alejandro Silva-Palacios, Gabriela Navarrete-Anastasio, Elizabeth Lira-Silva, Leonardo del Valle-Mondragón, Willy Ramsés García-Niño, Angélica Ruiz-Ramírez, Francisco Correa, Francisco Javier Roldán-Gómez, Jesús Vargas-Barrón, Alberto Aranda, Salvador Uribe-Carvajal, Natalia Pavón

**Affiliations:** 1 Department of Cardiovascular Biomedicine, Instituto Nacional de Cardiología Ignacio Chávez, Mexico City 14080, Mexico; 2 Department of Pharmacology, Instituto Nacional de Cardiología Ignacio Chávez, Mexico City 14080, Mexico; 3 Outpatient Department, Instituto Nacional de Cardiología Ignacio Chávez, Mexico City 14080, Mexico; 4 Research Coordination, Instituto Nacional de Cardiología Ignacio Chávez, Mexico City 14080, Mexico; 5 Department of Pathological Anatomy, Instituto Nacional de Cardiología Ignacio Chávez, Mexico City 14080, Mexico; 6 Department of Molecular Genetics, Institute of Cellular Physiology, Universidad Nacional Autónoma de México, Mexico City 04510, Mexico

**Keywords:** menopause, EDMs, 2-hydroxyestrone, 17β-estradiol-3-methyl-ether, cardiomyocytes, cardiac function, oxidative stress

## Abstract

**Background**: For a long time, it was thought that estrogen degradation metabolites (EDMs) had no role in the female body, but this view has changed. Despite the progress, there are still unknowns about EDMs and the heart. We found that EDMs affect isolated cardiac mitochondria, which may impact heart function. Therefore, our objective was to explore the effect of two EDMs, Met A (2-hydroxyestrone) and Met D (17β-estradiol-3-methyl-ether), on two experimental models: rat H9c2 cardiomyoblast cells exposed for increasing times to different concentrations of each EDM, and, also, the effects of each metabolite on heart function in intact and oophorectomized (Oopho) rats. **Methods**: The cell viability, mitochondrial potential, and oxidative stress were assayed in H9c2 cells. Echocardiographic analyses, histological analyses, and an assessment of cardiac damage and oxidative stress in intact and Oopho rats were tested. **Results**: The strongest negative effects were seen with Met D, where antioxidant capacity decreased and oxidative stress increased significantly. Upon further analysis of the effects of each metabolite on heart function, echocardiographic analysis revealed that cardiac function had not changed in either group, although clear signs of cardiac hypertrophy and fibrosis were observed. **Conclusions**: Our results suggest EDMs have specific effects on female rats. This study emphasizes the need to thoroughly investigate EDMs’ side effects and impact on the heart.

## 1. Introduction

Estrogen metabolism involves the interconversion of 17β-estradiol (E2) and estrone (E1), as well as by cytochrome P450 (CYP)-mediated conjugation and deconjugation reactions, glucuronidation, O-methylation, sulfation, and oxidation (primarily hydroxylation). Hydroxylation is the first irreversible step in estrogen metabolism mediated by CYP [1]. These reactions generate many byproducts known as estrogen degradation metabolites (EDMs) [2].

Some EDMs exhibit effects unlike those of parent hormones (estrogens) and, thus, act as endocrine disruptors [3]. Indeed, the previous data suggest that EDMs may disrupt cardiac function [4]. Most CYP enzymes are abundant in the liver: estrogen metabolism occurs in this organ, where approximately 80% of estradiol is transformed to 2-hydroxyestradiol and 20% to 4-hydroxyestradiol. These products generate free radicals through an oxidation–reduction cycle involving semiquinone and quinone derivatives that damage DNA. Endometrial adenocarcinoma is induced by 4-hydroxyestradiol [5] and this metabolite has been detected in human uterine fibroids and breast tumors [6]. Despite the above, the potential effects of EDMs on other organs have not yet been well-characterized.

Gaikwad et al. [7] noted that, in postmenopausal women with breast cancer, up to 40 different metabolites were elevated in urine as compared to healthy women. Conversely, antioxidant and cardioprotective effects of 2- and 4-hydroxyestradiol have been reported in the adriamycin-induced cardiomyopathy of oophorectomized (Oopho) rats [8]. Indeed, we reported that menopausal women with cardiovascular disease have an increase in at least 10 metabolites as healthy menopausal women [4].

To investigate EDMs’ effects on the heart, we examined two metabolites. First, we studied 2-hydroxyestrone (Met A), a.k.a. “the good metabolite”, as it inhibits the growth and proliferation of breast cancer cells [9] and exhibits antioxidant effects [10]. Then, we studied 17β-estradiol-3-methyl-ether (Met D), which has early toxic effects on isolated cardiac mitochondria [2]. Our aim was to determine how these EDMs affect the viability of H9c2 cardiomyocytes and cardiac function in Oopho and in control rats.

## 2. Materials and Methods

### 2.1. Cell Culture Conditions and Treatments

The H9c2c (2-1) cell line (CRL-1446™) was kindly provided by Dr. Rebeca López-Marure (Instituto Nacional de Cardiologia Ignacio Chávez, Mexico City, Mexico). H9c2 cell line is a subclone of the original cell line derived from embryonic BD1X rat (*Rattus norvegicus*) heart tissue and exhibits many of the properties of skeletal muscle, and was purchased from ATCC^®^ (Manassas, VA, USA) and grown in DMEM (Gibco, Grand Island, NY, USA) with 10% fetal bovine serum (FBS, Biowest, Riverside, MO, USA) and 1% antimycotic–antibiotic solution Gibco, Grand Island, NY, USA), at 37 °C with 5% CO_2_. The final concentrations of Met A and Met D were set after exposing 3 × 10^3^ cells/well to different concentrations from a 1 ng/mL stock solution. After treatment, the cells were incubated with 12 μM fluorescein diacetate (FDA, Sigma-Aldrich, St. Louis, MO, USA) for 5 min at 37 °C in the dark. Fluorescence intensity was quantified using a Biotek Synergy HT multimodal plate reader (Agilent, Santa Clara, CA, USA) with 452 nm and 522 nm excitation/emission filter sets, respectively. Cell viability was expressed as a percentage and normalized to that of the not-treated group (NT).

### 2.2. Mitochondrial Transmembrane Potential

Briefly, 3 × 10^3^ cells/well were seeded into 96-well plates, washed twice with PBS, and incubated with 100 nM tetramethylrhodamine (TMRE) for 20 min at RT. After two washes, cells were incubated with DMEM and placed on a Biotek Sinergy HTX multimodal fluorescence plate reader (Agilent Technologies, Santa Clara, CA, USA) at 545/600 nm. Fluorescence data were normalized to the NT group.

### 2.3. 2′,7′-Dichlorodihydrofluorescein Diacetate (DCFH-DA) Measurement

To assess oxidative stress, 3 × 10^3^ cells were seeded in 96-well plates and incubated with 10 μM DCFH-DA for 30 min at 37 °C in the dark [11]. After two washes with DMEM and one wash with PBS, the cells were observed in the green fluorescent protein (GFP) channel and acquired using a Lionheart FX automated live-imaging microscope (Agilent, Santa Clara, CA, USA) and the values were normalized to those of the NT group.

### 2.4. Animals and Experimental Groups

The size of the sample was calculated using the formulae from previous reports [12]. Thirty female Wistar rats (weighing between 180 and 200 g) were used in the experiments and randomized into intact rats (*n* = 15) and Oopho rats (*n* = 15). Oophorectomy was performed under anesthesia with pentobarbital (25 mg/100 g), as described previously [13]. After surgery, rats were housed in our animal colony and maintained under controlled light//dark cycles (12 h each) with water and rodent chow ad libitum until they were 6 months of age. After that, the rats (intact and Oopho) were randomly selected and divided into three groups (*n* = 5 each): solution saline isotonic (SSI), Met A, and Met D. The experiments were conducted in agreement with ethical rules and guidelines from the National Institute of Cardiology Ignacio Chávez, México, Record Num 14-865.

### 2.5. Echocardiography (Echo) Analysis

Intact and Oopho rats were anesthetized with sodium pentobarbital (60 mg/kg; i.p.) Under deep anesthesia, a midline incision was made along the neck, followed by tracheostomy. Animals were mechanically ventilated with low-flow oxygen using a small-species ventilator (Ugo Basile, Gemonio (VA), Italy) at a rate of 70 breaths/min. After left thoracotomy and heart exposure, 2D images of the cardiac chamber were obtained using the epicardial approach from a long-axis view of the left ventricle (LV) at the papillary muscle level using a SONOS 550 echocardiographer (Koninlijke Phillips Electronics, Eindhoven, The Netherlands) with a 12 MHz transducer. After a 5 min stabilization period, baseline echo was recorded. Then, SSI, Met A (100 ng), or Met D (100 ng) was injected into the LV’s free wall, each in a 100 μL volume, to perform another echo after 10 min. Parameters like LV internal diameter, LV posterior wall thickness, fractional shortening (FS), and ejection fractions (EF) were calculated from the LV dimensions as per our previous report [14]. After cardiac evaluation, the animals were euthanized via cardiectomy. Lungs and hearts were weighed, and the tibial length (TL) of each rat was measured to calculate the ratio between the two organs. Lastly, the hearts were preserved as planned in the experiment.

### 2.6. Cardiac Injury Markers

Blood was drawn after the echocardiography, collected in EDTA tubes, and centrifuged to obtain plasma. Plasma was used to measure lactate dehydrogenase (LDH, BioSystems, Barcelona, Spain) and creatine kinase-MB (CK-MB, Randox. Laboratories, Crumlin, UK) activities, as described by the manufacturer.

### 2.7. Cardiac Histology

A section of the heart was fixed by immersion in 4% formaldehyde in PBS, embedded in paraffin, and sectioned (5 μm thick) using a Leica RM 2125RT rotary microtome (Leica Biosystems, Wetzlar, Germany). Sections were stained with standard hematoxylin and eosin (H&E) or Masson’s trichrome. The fields from three independent tissue sections were examined in accordance with our previous reports using a Lionheart FX automated live-imaging microscope (Agilent, Santa Clara, CA, USA) at 40× magnification [15].

### 2.8. Immunofluorescence

Five-micrometer-thick tissue sections were placed on electrocharged slides, permeabilized with 0.1% PBS-Triton X-100 for 30 min and then neutralized with 50 mM ammonium chloride for 10 min. After retrieving antigens using 0.3 M sodium citrate and 3 M NaCl at pH 6.9 and 60 °C for 15 min, and blocking step with 5% BSA for 1 h, the slides were incubated with CK-MB antibody (1:250; Santa Cruz Biotechnology, Dallas, TX, USA) overnight in a humidified chamber at 4 °C. The slides were then incubated with a secondary antibody (1:1000; Alexa Fluor 488, Abcam, Cambridge, UK) for 2 h at RT and washed thrice with PBS. The nuclei were stained with DAPI (1:5000; Sigma-Aldrich, St. Louis, MO, USA) for 3 min in PBS, and the slides were mounted using an aqueous mounting medium (Santa Cruz Biotechnology, Dallas, TX, USA). At least five independent fields from three different sections were analyzed using a Lionheart FX automated live-imaging microscope (Agilent, Santa Clara, CA, USA) at 40× magnification.

### 2.9. Oxidative Stress Markers

Markers of oxidative stress and antioxidant enzymes were measured in the cell culture medium recovered after treating the cells with the metabolites and in the plasma from rats treated with both metabolites.

#### 2.9.1. Total Antioxidant Capacity (TAC)

TAC was quantified using a spectrophotometric method. In brief, the samples were analyzed in the UV–Vis region at room temperature (Cary 4000, Varian Inc., Mulgrave, VIC, Australia) at 720 nm. The equipment was adjusted with a reagent blank. TAC concentration was determined using a standard curve from 0–1500 µmol analytical grade ferrous sulfate/L [16].

#### 2.9.2. Malondialdehyde and Malonate

Malondialdehyde (MDA) and malonate were determined simultaneously in treated samples by capillary zone electrophoresis [17]. MDA and malonate concentrations were (pmol/mL) and determined using a standard curve.

#### 2.9.3. 8-Hydroxy-2-Deoxyguanosine (8HO2dG)

8HO2dG was determined in the treated samples by capillary zone electrophoresis and UV detection using a diode array, according to previous reports [18]. The concentration of 8HO2dG was determined using a standard curve and the results are expressed in pmol/mL.

### 2.10. Statistical Analysis

GraphPad Prism software (version 10.0; San Diego, CA, USA) was used for graphical presentation and statistical analysis. Results are expressed as mean ± standard error (SE). Comparisons between treatments (SS, Met A, or Met D) were analyzed separately in intact or Oopho rats and at baseline or post-treatment, using two-way ANOVA followed by Fisher’s LSD post hoc test. To assess differences between intact and Oopho rats, a non-paired *t*-test was used, stratifying animals by treatment (SS, Met A, or Met D) or time of treatment (baseline or post-treatment). Finally, the impact of treatment was evaluated by comparing baseline and post-treatment values using a paired *t*-test for each treatment and intervention group. All *p*-values < 0.05 were considered statistically significant. The sample size was determined based on previously published experiments in which statistical differences were observed [13].

## 3. Results

### 3.1. Exposure to Either Metabolite Affects Cell Viability

**Figure 1:**
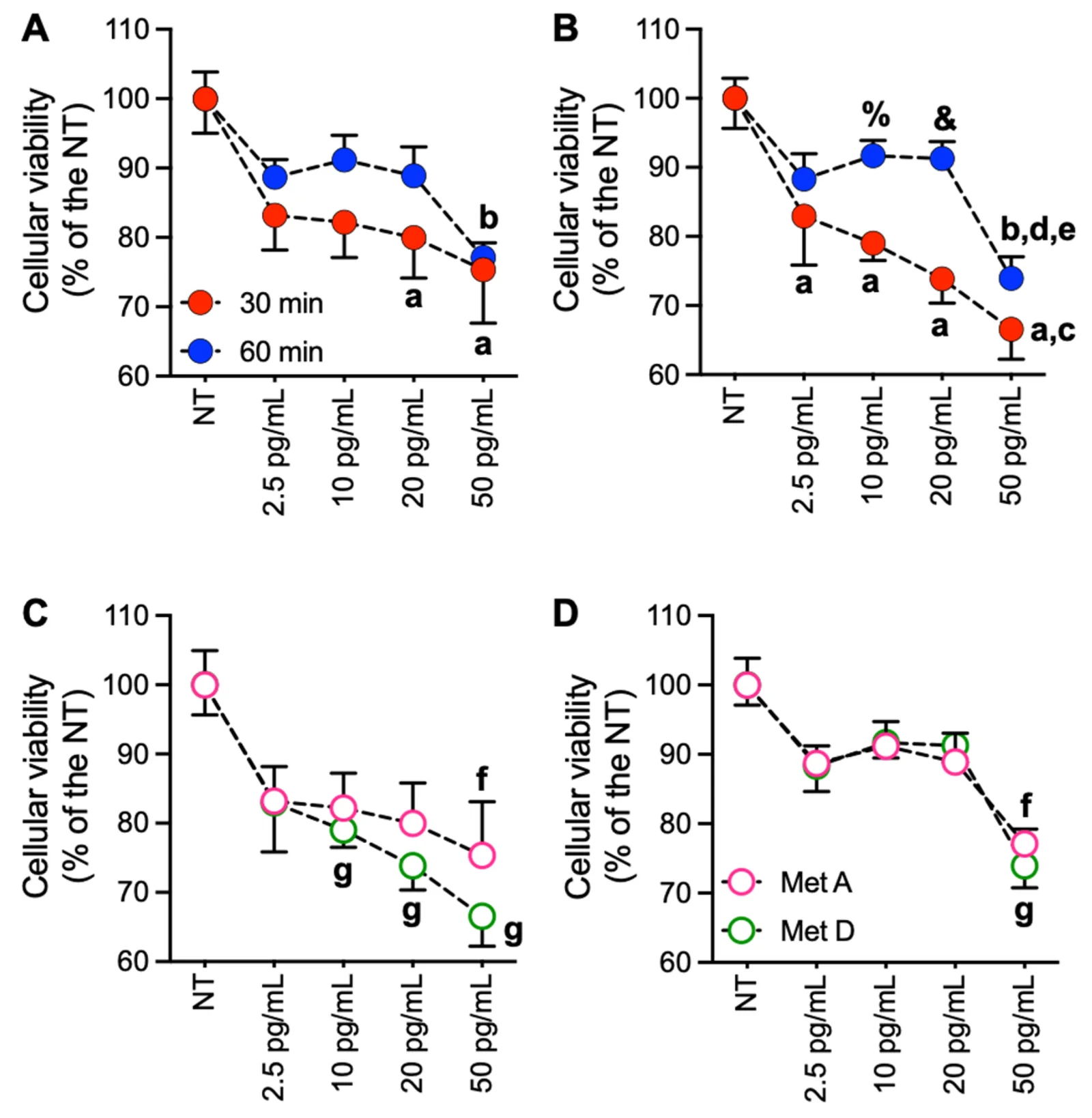
Viability of H9c2 cells exposed to Met A and Met D. Cell viability was assessed using fluorescein diacetate (FDA; *n* = 3 in triplicate) by incubating different concentrations of (**A**) Met A or (**B**) Met D for 30 min (red circle) or 60 min (blue circle). Comparison of incubation times: (**C**) 30 min and (**D**) 60 min with Met A (pink circles) and Met D (green circles). The results are presented as the mean ± SE. ^a^ *p* ≤ 0.05 vs. NT 30 min; ^b^ *p* ≤ 0.05 vs. NT 60 min; ^c^ *p* ≤ 0.05 vs. 2.5 pg/mL 30 min; ^d^ *p* ≤ 0.05 vs. 10 pg/mL 60 min; ^e^ *p* ≤ 0.05 vs. 20 pg/mL 60 min. ^%^ *p* ≤ 0.05 vs. 30 min; ^&^ *p* ≤ 0.05 vs. 30 min. For panel (**C**,**D**): ^f^ *p* ≤ 0.05 vs. NT Met A; ^g^ *p* ≤ 0.05 vs. NT Met D. NT: Non-treated. For clarity, viability data on the Y axis are cut at 60%.

We analyzed the effect of increasing concentrations of each metabolite on the viability of cardiomyoblasts, a cell line exhibiting a non-contractile cardiomyocyte phenotype. As shown in Figure 1A, Met A reduced cell viability after just 30 min of exposure. At a concentration of 50 pg/mL, cell viability dropped by up to 25% at both time points compared to the NT group. Similarly, after 30 min with Met D, cell viability decreased in a dose-dependent manner (Figure 1B). When comparing both metabolites at the same exposure time, Met D was more harmful to the cells at doses between 10 and 50 pg/mL than Met A, which was toxic only at the highest dose (Figure 1C). For the 60 min exposure (Figure 1D), both metabolites showed similar effects, where 50 pg/mL was the most harmful.

### 3.2. Met D Induces Higher Oxidative Damage in H9c2 Cells

We studied whether decreased cell viability was linked to oxidative stress by examining various markers. Although 30 min of exposure to Met A did not increase the reactive oxygen species (ROS) levels, the transmembrane potential decreased at all tested concentrations (Table 1), indicating mitochondrial dysfunction. The MDA levels remained unchanged; in contrast, 8OH2dG rose in cells exposed to 2.5 and 10 pg Met A/mL compared with the NT group. Oddly, TAC was significantly higher at 2.5 pg/mL, with a decrease at the remaining concentrations, with no differences from the NT group (Table 1). Meanwhile, ROS production was higher at 20 pg Met D/mL compared to its control. In contrast, the transmembrane potential decreased in all conditions, leading to marked lipid peroxidation. Meanwhile, 8OH2dG was lower at 2.5 pg Met D/mL, with no changes in TAC and malonate levels (Table 1). After 60 min of incubation, oxidative damage did not increase, even at 50 pg Met A/mL (Table 1). Simultaneously, ROS levels increased to 20 pg/mL, MDA at 10 pg/mL, and 8OH2dG at 2.5 pg/mL with Met D at this same time, even though TAC was higher at 2.5 pg/mL compared to the NT group. Finally, when comparing the effect of each concentration on each metabolite, we observed that Met D induced greater lipid peroxidation than Met A. Concentrations of 2.5 and 10 pg/mL of Met D decreased the TAC and malonate levels, while raising the MDA levels compared to Met A, regardless of the time point (Table 1). Overall, despite the differences in concentrations and time points, increased oxidative damage was associated with reduced cell viability (Figure 1).

### 3.3. Morphometric Parameters Remain Unchanged in Oopho Rats Exposed to EDMs

After confirming the metabolite’s in vitro effects, with Met D inducing the highest cytotoxicity at both time points, we tested the effects of both EDMs in a rat model using intact and Oopho rats. The morphometric changes are summarized in Table 2. Oopho rats had a higher body weight (BW; 362.5 ± 13.2 g) than intact rats (246.4 ± 7.1 g) and this difference was also seen in the tibial length (5.4 ± 0.07 cm vs. 4.6 ± 0.04 cm). These changes were associated with an increase in the heart weight in Oopho rats (HW; 0.86 ± 0.03 g) compared to intact rats (0.77 ± 0.01 g), but with no changes in the lung weight (LW). Despite these changes, the ratio of HW to BW (HW/BW) was lower than that in the intact group, and the LW/BW ratio did not show any significant increase. Similarly, the HW/TL and LW/TL ratios did not change in the Oopho rats (Table 2). Following the treatment with Met A and Met D in Oopho rats, the evaluated parameters were not statistically significant compared to intact rats receiving the same treatments. Additionally, there were no significant differences between the Met A and Met D groups and those exposed to SSI in Oopho rats. The results indicated that the health status of rats treated with EDMs was not affected at this level.

### 3.4. Cardiac Function in Oopho Rats Exposed to EDMs

Representative images of the echocardiographic analysis in the long-axis M-mode (Supplementary Figure S1) indicated that the left ventricular diastolic (d) and systolic (s) diameters (LVED) were larger in Oopho rats than in intact rats (though without significant changes), with preserved EF and FS before SSI administration. Thereafter, LVPW, LVEDs, and HR increased in Oopho rats, although EF and SF did not (Table 3). In intact rats treated with Met A, only the LVPW thickness showed significant changes compared with the baseline values in intact rats. In addition, HR was reduced in rats treated with Met D, but there were no changes in EF. Our data suggest that neither of the metabolites alters the cardiac function in intact rats. Furthermore, LVEDd and LVEDs were greater in Oopho rats given Met A than in intact rats exposed to the same metabolite, while LVPW in this group exceeded the baseline value in Oopho rats (Table 3). Following Met D injection, LVEDd and LVEDs increased in the Oopho + Met D group (Table 3). %EF was lower in the Oopho + Met D group (79.9 ± 3.9%) compared with the Oopho Baseline + SS group (85.5 ± 2.6%); however, when comparing this parameter with that of the Oopho rats in the Baseline group without Met D, EF remained unchanged (77.3 ± 4.7%) (Table 3).

### 3.5. Histopathological Changes in Oopho Rats Exposed to EDMs

**Figure 2:**
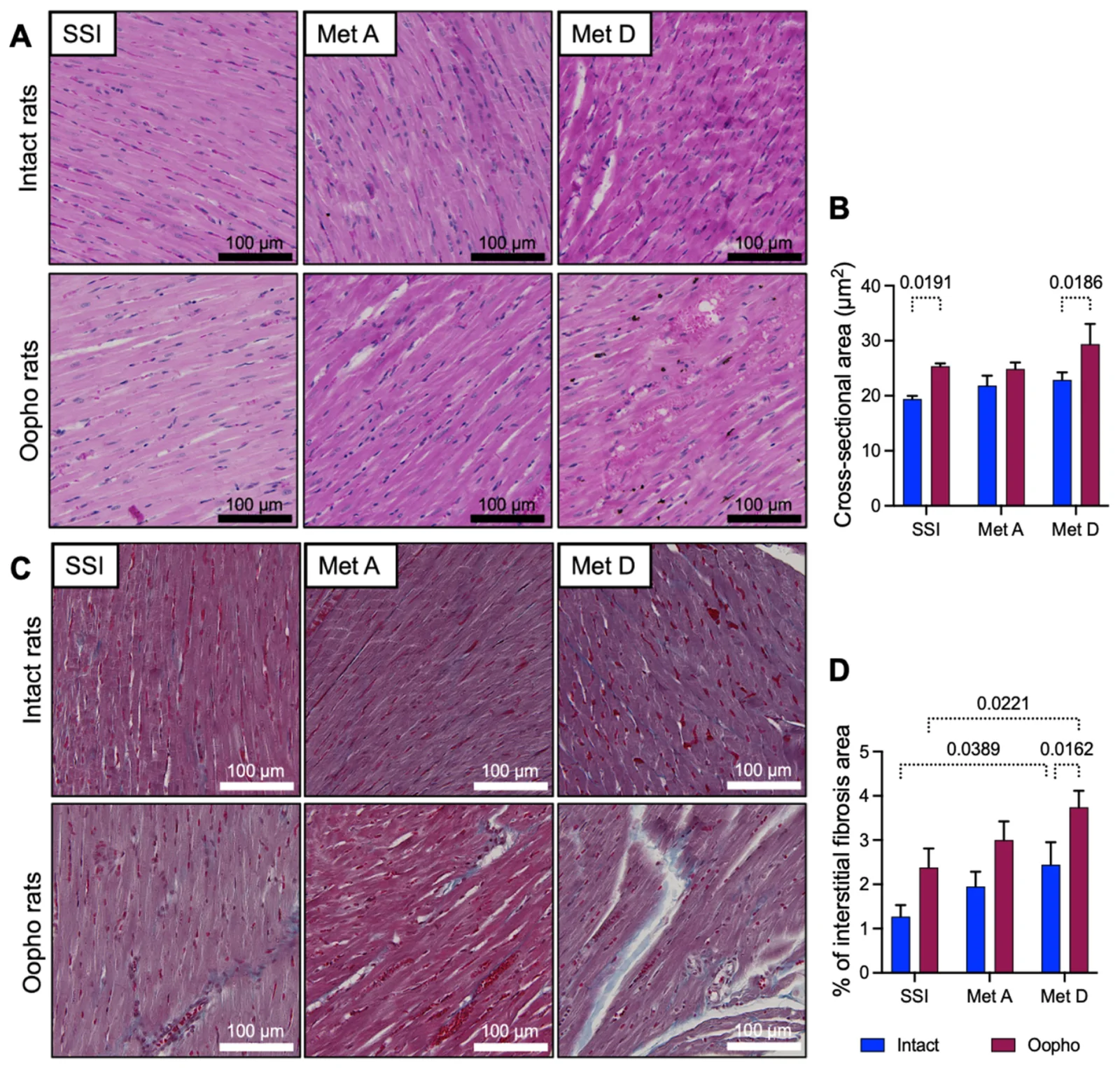
Changes in cardiac tissue from Intact and Oopho rats injected with EDMs. Representative images of cardiac tissue stained with hematoxylin and eosin (H&E) are shown in panel (**A**) and in (**C**) stained with Masson’s trichrome. The left ventricular free wall was analyzed and captured using an image analyzer at 40× magnification (bar scale: 100 μm, *n* = 3; five random fields were analyzed per sample). In (**B**), the cross-sectional area of cardiomyocytes, and, in (**D**), the area of interstitial fibrosis corresponding to panels (**A**) and (**C**) are plotted, respectively. The results are shown as the mean ± SE. SSI: Saline solution isotonic; Met: Metabolite.

Using EDMs as an intervention could lead to possible confounding factors such as local myocardial injury and post-injection trauma. Supplementary Figure S2 illustrates the site of administration and analysis to prevent any misinterpretations in our study. Considering this, we observed that the Intact + SSI group exhibited a myocardium with a preserved morphology, with compact and well-organized muscle fibers, without signs of cellular damage (Figure 2A). No morphological changes (Figure 2A) or altered fibrosis (Figure 2C,D) were noted in the Oopho + SSI group and a slight enlargement of cardiomyocytes was observed (Figure 2B). In intact rats injected with Met A, mild hypereosinophilia was observed without any structural changes (Figure 2A), whereas the Oopho + Met A group showed a slight disorganization of cardiac fibers (Figure 2A), not linked to hypertrophy or fibrosis (Figure 2B,D). The Intact + Met D group exhibited greater hypereosinophilia and fiber disorganization (Figure 2A) related to changes in cardiomyocyte size (Figure 2B), and a significant increase in fibrosis (Figure 2C,D). This effect was most evident in the Oopho + Met D group, which had clear fiber disorganization and vacuolar degeneration with a fine, diffuse microvesiculation of cardiomyocytes (Figure 2A), associated with greater hypertrophy and fibrosis (Figure 2B,D) compared to Intact + Met D and Intact + SSI rats, respectively (Figure 2B).

### 3.6. Cardiac Markers in Oopho Rats Exposed to EDMs

**Figure 3:**
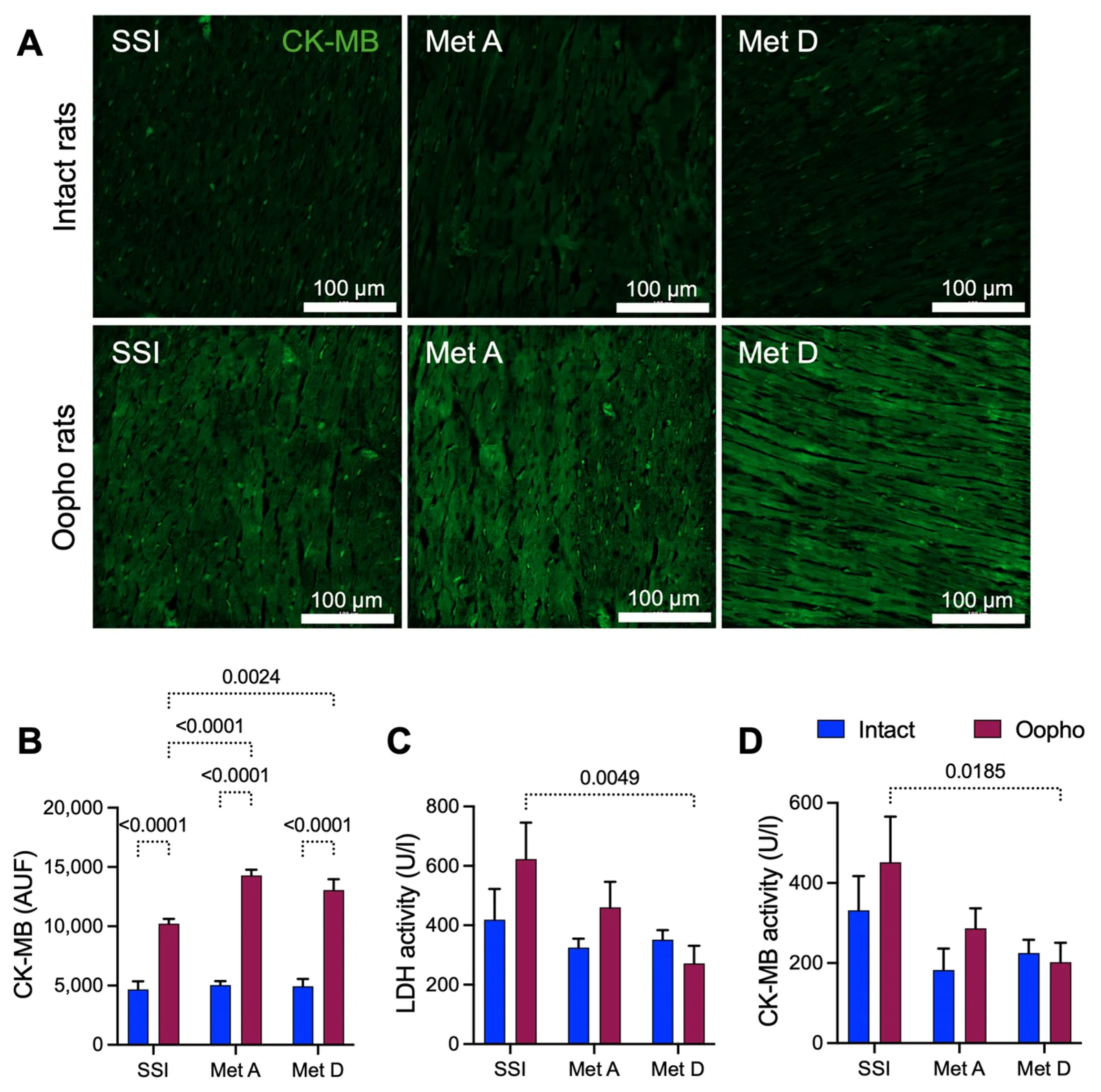
Cardiac damage in Intact and Oopho rats injected with EDMs. (**A**) Transverse section of LV immunostained with CK-MB. The slices (*n* = 3) were analyzed and captured using an image analyzer at 40× magnification (bar scale: 100 μm, five random fields were analyzed per sample). (**B**) Quantitative analysis of fluorescence intensity, reported as arbitrary units of fluorescence (AUF). Evaluation of (**C**) creatine kinase myocardial band (CK-MB) and (**D**) lactate dehydrogenase (LDH) in the serum of rats (*n* = 5 independent samples). The results are shown as the mean ± SE. SSI: Saline solution isotonic; Met: Metabolite.

We analyzed the expression of CK-MB in the cardiac tissue of intact and Oopho rats as a key biomarker of tissue injury (Figure 3A). In the first group, the metabolites did not change the CK-MB expression (Figure 3B). In contrast, in the Oopho rats, the CK-MB levels nearly doubled compared to the Intact + SSI group, and this rise persisted after the injection of both metabolites (Figure 3B). Additionally, we assessed the LDH (Figure 3C) and CK-MB (Figure 3D) levels in the plasma of all experimental groups. The activity of these enzymes was lower in the Oopho + Met D group (Figure 3C,D).

### 3.7. Changes in Oxidative Stress and Antioxidant Markers in Oopho Rats Exposed to EDMs

We assessed markers of oxidative stress and antioxidant activity in vivo (Table 4). Compared to intact rats, Oopho rats showed a threefold increase in malonate activity, which dropped after Met A exposure. Met D had stronger effects on intact rats than Met A. The levels of NO, TAC, and BH4 went down, while BH2 levels increased fourfold. Oddly, Met A promoted marked changes in Oopho rats than in intact rats with the same metabolite; that is, NO and BH4 activity decreased, whereas BH2 levels remained elevated. In contrast, compared with Oopho rats treated with SSI, Met A increased MDA and reduced malonate activity. Paradoxically, Met D in Oopho rats did not induce changes in any of the evaluated markers (Table 4).

In summary, the reduction in viability in H9c2 cardiomyoblasts was associated with alterations in parameters related to oxidative stress—including mitochondrial membrane depolarization, ROS production, lipid peroxidation, and DNA damage. The response depends on the specific metabolite, concentration, and exposure time. Furthermore, in the in vivo model, we determined that EDMs do not produce detectable changes in the morphometric status of Oopho rats but do promote limited functional cardiac changes that, interestingly, were not linked to significant alterations in oxidative stress.

## 4. Discussion

In a previous study, we found that at least 10 different metabolite concentrations differed between healthy menopausal women and those at risk for or already suffering from cardiovascular disease. Some of these metabolites showed different effects on cardiac mitochondria [2]. In this study, we examined the differential effects of two metabolites, Met A (2-hydroxyestrone) and Met D (17β-estradiol-3-methyl-ether), in two models: cardiomyoblasts and Oopho rats. These findings provide further evidence of the important role of metabolites in the heart, besides their role in diseases such as cancer.

There is an increasing interest in how EDMs affect the cardiovascular system and related diseases. A groundbreaking study by Grohé et al. [19] found that 2-hydroxyestrone (1 nM for 24 h) significantly stimulates cardiac fibroblast growth, while 17β-estradiol (1 nM for 24 h) decreases L-type calcium (Ca^2+^) currents by up to 70% in cardiomyocytes. Our data showed that, after 30 min of exposure, 2-hydroxyestrone and 17β-estradiol-3-methyl-ether at 2.5 to 50 pg/mL can decrease the viability of H9c2 cardiomyoblasts. Yet, this effect was less noticeable after 60 min (Figure 1). This suggests a possible time dependence, especially in women, whose metabolisms interact with these metabolites for years. In this scenery, it is especially relevant to consider that our study is among the first ones to explore the time of exposure to EDMs. There is a real possibility that the effects of systemic exposure could not be reached since we only used some hours and this leaves open the possibility to work with the effects in the long term; additional studies are needed in this regard.

In an immortalized human hepatocyte cell line, 2-hydroxyestrone (5 μM for 24 h) protects against ferroptosis. It also aids in acetaminophen-induced liver injury by decreasing ROS production and lipid peroxidation. This is achieved by inhibiting protein disulfide isomerase, which reduces the dimerization of inducible nitric oxide synthase (iNOS) [20]. These findings further show that organs’ susceptibility or resistance to EDMs depends on time and concentration.

The individual roles of each EDMs in the heart remain unknown. Some studies have shown how metabolites influence the heart through their receptors or estrogen-related receptors (EERs). For instance, 17β-estradiol (10^−7^ M) can cause arrhythmia by changing Ca^2+^ handling in female cardiomyocytes, but this effect disappears in an ERβ knockout mouse model [21]. Cardiac metabolism is also controlled by EER; using two agonists, the ɑ and ɣ isoforms of EERs, can improve EF, reduce fibrosis, and increase survival in pressure-overload-induced heart failure (HF) [22].

In Oopho rats, 17-estradiol was particularly low, leading to significant changes in IVS thickness and LV mass, and a slight reduction in EF without signs of cardiac hypertrophy [23]. Our study is the first to examine the direct effects of two EDMs on cardiac function in Intact and Oopho rats (Table 3). Although cardiac function was not impaired, early remodeling processes like hypertrophy and fibrosis were observed (Figure 2 and Figure 3), suggesting a compensatory mechanism to preserve heart function. However, this effect is not always observed in Oopho rats, as recent studies show that the heart function and remodeling remain unchanged. Interestingly, this changes when Oopho rats develop diabetic cardiomyopathy, which enhances inflammasome activation and fibrosis [24].

The decline in estrogen levels following menopause promotes low-grade systemic inflammation and a local pro-inflammatory environment, thereby exacerbating endothelial dysfunction. This scenario leads to impaired diastolic function and more pronounced myocardial fibrosis–factors that, together, drive the development of heart failure (HF) [25]. For example, using a loss-of-function mutation in estrogen receptor alfa (ERα), it was demonstrated that females exhibit right ventricular-vascular uncoupling, diastolic dysfunction, and fibrosis following pressure overload. Furthermore, potential mediators of ERα-regulated pathways were identified, such as metallopeptidase inhibitor 1, matrix metalloproteinase 9, kallikrein-related peptidase 10, and Jun proto-oncogene [26].

A high-fat diet combined with Nω-nitro-L-arginine methyl ester (L-NAME) in an HFpEF C57BL/6J mouse model leads to a greater left ventricle EF, a smaller rise in E/e′, and insignificant changes in HW and the left ventricle remodeling index after 5 weeks of treatment compared to male mice [27,28]. Oddly, an ovariectomized female on this diet for 15 weeks showed a heart weight and diastolic function like a Sham group, suggesting that the reduced remodeling response in female mice is likely not influenced by female sex hormones [27]. Considering these variations, it has been proposed mechanistically that males typically exhibit a higher mitochondrial DNA content and activity than females, partially controlled by sex hormones. RNA-seq analysis found that acyl-CoA synthetase long-chain family member 6 is a key downstream factor of sex hormones that controls mitochondrial activity and the metabolic changes responsible for sex bias [28].

Estrogen deficiency led to oxidative stress and decreased nitric oxide (NO) production [29]. Our data indicate that oxidative stress increases in H9c2 cells exposed to 2-hydroxyestrone and 17β-estradiol-3-methyl-ether, and the effects vary with exposure time (Table 1), while the effects were more pronounced with 2-hydroxyestrone in cardiac tissue (Table 4). To counteract this damage, cells activate the antioxidant response, which may offer protection against oxidative stress during menopause, similar to other age-related conditions [30]. For instance, tempol supplementation (a superoxide scavenger) significantly improved plasma oxidative stress in Oopho rats [23]. We found that Oopho rats tend to have a lower antioxidant capacity than intact rats, although none of these metabolites altered this pattern. When the cellular BH4/BH2 ratio drops, eNOS uncoupling increases, leading to a cycle of oxidative stress [31]. In our study, Oopho rats did not exhibit NO uncoupling, as their values remain unchanged before or after the metabolite injection. Surprisingly, BH4 levels were higher in intact rats treated with Met A, suggesting greater oxidative stress (Table 4) and possible pleiotropic effects of EDMs under physiological conditions.

Our data suggest an increase in oxidative stress and mitochondrial dysfunction following Met D treatment, which could stem from the activation of other cellular processes triggering this state. Specifically, this could be associated with inefficiencies in the electron transport chain (e.g., mitochondrial complexes I, III, and IV) and ROS production. Furthermore, the downregulation of signaling cascades cannot be ruled out. In this regard, a key pathway involved in the regulating cellular redox balance is the Nrf2 transcription factor, which can upregulate the gene expression of antioxidant enzymes (e.g., heme-oxygenase-1, and NQO1) when the redox status is compromised [32]. On the other hand, mitochondrial ROS have been reported to act as second messengers, activating pathways such as the NLRP3 inflammasome and pro-inflammatory routes orchestrated by the NF-kB transcription factor [33]. Consequently, monitoring these pathways would provide a more comprehensive insight into the mechanism of action of EDMs.

Research using Oopho models has connected several mechanisms to a hormone deficiency, including mitochondrial dysfunction and impaired mitophagy [2,24,34]. In this regard, it is well-established that sex hormones, such as estrogen, play a direct regulatory role in cellular energy production, antioxidant defense, and mitochondrial quality control [35]. Furthermore, this organelle regulates steroidogenesis in ovarian cells, as it houses steroidogenic enzymes (e.g., P450scc) that convert cholesterol into precursor hormones such as pregnenolone [36]. When mitochondrial damage occurs and mitophagy fails, toxic ROS accumulate, energy production declines, and hormone-producing cells undergo premature cellular senescence or death [37]—processes that collectively promote further damage induced by EDMs [2]. Our study could be a starting point for exploring the detailed mechanisms and other cellular processes involved in the action of EDMs in the female body. In this context, the influence of estrogens on myocardial post-translational modifications and heart function in women has been reviewed [38].

## 5. Conclusions and Limitations of Our Study

Although there is still much to learn about the role of EDMs in the heart, our data revealed the differential effects of EDMs in female rats, emphasizing the need to thoroughly study their side effects and impact on the heart. Our study is exploratory in nature and is based on an acute local intervention with EDMs; therefore, this scenario may not adequately replicate the systemic exposure to estrogen metabolites or the long-term effects of menopause; it nevertheless highlights the need to intensify the analysis of the side effects of EDMs on the heart in future research.

## Figures and Tables

**Table 1:** Oxidative markers assessed in culture medium from H9c2 cells exposed to Met A and Met D.

		Met A (pg/mL)					Met D (pg/mL)				
Time	Marker	NT	2.5	10	20	50	NT	2.5	10	20	50
30 min	DCFH (% of baseline)	1 ± 0.0	0.97 ± 0.02	0.95 ± 0.02	0.89 ± 0.04	1.05 ± 0.02 ^@^	1 ± 0.0	1.02 ± 0.007	1.04 ± 0.04	1.27 ± 0.12 ^d,#,&,$^	1.15 ± 0.09
	TMRE (% of baseline)	1 ± 0.0	0.59 ± 0.05 *	0.52 ± 0.02 *	0.47 ± 0.03 *	0.41 ± 0.02 *^,@^	1 ± 0.0	0.63 ± 0.08 ^#^	0.57 ± 0.08 ^#^	0.53 ± 0.05 ^#^	0.49 ± 0.04 ^#^
	MDA (pmoles/mL)	18.2 ± 3.1	8.9 ± 2.9	9.9 ± 1.5	9.8 ± 2.2	25.4 ± 2.7	18.2 ± 3.1	51.5 ± 6.4 ^b,#^	66.4 ± 15.6 ^#,&,c^	54.2 ± 5 ^#,$,d^	64.4 ± 12.2 ^#,$,e^
	8HO2dG (pM)	0.1 ± 0.03	0.68 ± 0.3 *	0.67 ± 0.3 *	0.44 ± 0.2	0.11 ± 0.07 ^%,@^	0.07 ± 0.05	0.01 ± 0.004 ^b^	0.33 ± 0.19	0.01 ± 0.001	0.008 ± 0.002
	TAC (mmol/L)	33.6 ± 5.6	81.4 ± 15.7 *	58.5 ± 9.1	52 ± 13.5	38 ± 9.4 ^%^	21.1 ± 4.8	11.8 ± 1 ^b^	59 ± 21.5	12.3 ± 0.7 ^d^	12.9 ± 1.6
	Malonate (pmoles/mL)	5.7 ± 1	9.3 ± 2.3	10.6 ± 2.5	6.8 ± 0.9	4.9 ± 0.9 ^@^	2.4 ± 1.1	1.5 ± 0.2 ^b^	7.4 ± 3.8 ^&^	1.5 ± 0.1 ^$^	1.3 ± 0.3 ^$^
60 min	DCFH (% of baseline)	1 ± 0.0	1.06 ± 0.04	1.13 ± 0.04	1.37 ± 0.17 ^#^	1.26 ± 0.08	1 ± 0.0	1.007 ± 0.01	1.06 ± 0.07	1.32 ± 0.2 ^#^	1.26 ± 0.14
	TMRE (% of baseline)	1 ± 0.0	1.35 ± 0.25	1.3 ± 0.18	1.3 ± 0.17	1.56 ± 0.15	1 ± 0.0	1.12 ± 0.07	0.83 ± 0.07 ^c^	1.18 ± 0.1	0.54 ± 0.0 ^?,a^
	MDA (pmoles/mL)	10 ± 2.5	12.2 ± 2	9.6 ± 1.6	24.2 ± 3.7	18.4 ± 4.4	14.3 ± 5	10.3 ± 3.9	59.3 ± 15.2 ^c,#,&^	41.8 ± 12	32.5 ± 10 ^$^
	8HO2dG (pM)	0.56 ± 0.2	0.43 ± 0.2	0.53 ± 0.2	0.03 ± 0.005 *^,@^	0.43 ± 0.1	0.17 ± 0.1	0.44 ± 0.1 ^#^	0.03 ± 0.01 ^c,&^	0.21 ± 0.1	0.1 ± 0.04
	TAC (mmol/L)	70.8 ± 13	70.3 ± 17	83.6 ± 11.8	30.09 ± 3.3 *^,%,@^	56.9 ± 9.7	34 ± 11.4 ^a^	68.7 ± 20.2 ^#^	14.5 ± 1.6 ^c,&^	34.4 ± 13.2 ^&^	27.1 ± 7.2 ^&^
	Malonate (pmoles/mL)	7 ± 1.9	9.2 ± 1.7	8 ± 1.4	9.6 ± 6.3	8.6 ± 2.1	8.4 ± 2	9.2 ± 1.6	2.1 ± 0.2	6.5 ± 1.8	5.3 ± 1.3

Data are shown media ± SE of 5 independent experiments. * *p* ≤ 0.05 vs. Met A NT; ^#^ *p* ≤ 0.05 vs. Met D NT; ^%^ *p* ≤ 0.05 vs. Met A 2.5 pg/mL, ^@^ *p* ≤ 0.05 vs. Met A 10 pg/mL, ^&^ *p* ≤ 0.05 vs. Met D 2.5 pg/mL, ^$^ *p* ≤ 0.05 vs. Met D 10 pg/mL, ^?^ *p* ≤ 0.05 vs. Met D 50 pg/mL; ^a^ *p* ≤ 0.05 vs. Met A NT, ^b^ *p* ≤ 0.05 vs. Met A 2.5 pg/mL, ^c^ *p* ≤ 0.05 vs. Met A 10 pg/mL, ^d^ *p* ≤ 0.05 vs. Met A 20 pg/mL, ^e^ *p* ≤ 0.05 vs. Met A 50 pg/mL. Met: Metabolite; NT: Non-treated. DCFH: 2′,7′-dichlorodihydrofluorescein; TMRE: Tetramethylrhodamine; TAC: Total antioxidant capacity; MDA: Malondialdehyde; 8HO2dG: 8-Hydroxy-2-deoxyguanosine.

**Table 2:** Morphometric analysis from intact and ovariectomized (Oopho) rats.

	Intact Rats			Oopho Rats		
Parameter	SS	Met A	Met D	SS	Met A	Met D
BW (g)	246.4 ± 7.1	247 ± 4.2	246.3 ± 4.6	375.2 ± 13.2 ^a^	339.4 ± 13.9 ^b^	378 ± 15 ^c^
HW (g)	0.77 ± 0.01	0.73 ± 0.02	0.80 ± 0.03	0.86 ± 0.03 ^a^	0.87 ± 0.02 ^b^	0.85 ± 0.03
LW (g)	2 ± 0.15	1.86 ± 0.1	1.86 ± 0.1	1.6 ± 0.1	1.67 ± 0.2	1.72 ± 0.2
TL (cm)	4.6 ± 0.04	4.8 ± 0.05	4.8 ± 0.08	5.4 ± 0.07 ^a^	5.1 ± 0.05 ^b^	5.3 ± 0.09 ^c^
HW/BW (g/kg)	3.1 ± 0.09	2.9 ± 0.07	3.2 ± 0.1	2.3 ± 0.1 ^a^	2.5 ± 0.1 ^b^	2.2 ± 0.1 ^c^
LW/BW (g/kg)	8.1 ± 0.8	7.5 ± 0.5	7.5 ± 0.4	4.1 ± 0.2 ^a^	4.8 ± 0.6 ^b^	4.5 ± 0.5 ^c^
HW/TL (g/cm)	0.16 ± 0.005	0.15 ± 0.005	0.16 ± 0.009	0.16 ± 0.009	0.17 ± 0.003 ^b^	0.16 ± 0.004
LW/TL (g/cm)	0.43 ± 0.03	0.38 ± 0.02	0.38 ± 0.01	0.29 ± 0.02 ^a^	0.32 ± 0.05	0.32 ± 0.03

Data are shown media ± SE of 5 independent experiments. ^a^ *p* ≤ 0.05 vs. Intact SS; ^b^ *p* ≤ 0.05 vs. Intact Met A; ^c^ *p* ≤ 0.05 vs. Intact Met D. BW: Body weight; HW: Heart weight; LW: Lung weight; TL: Tibial length. SS: Saline solution, Met: Metabolite.

**Table 3:** Echocardiographic analysis from intact and ovariectomized (Oopho) rats.

	Intact Rats						Oopho Rats					
Parameter	Baseline	SS	Baseline	Met A	Baseline	Met D	Baseline	SS	Baseline	Met A	Baseline	Met D
IVS (mm)	19.6 ± 0.4	20.8 ± 0.7	21 ± 1.0	19 ± 0.3	19.6 ± 0.4	19.6 ± 0.8	20 ± 0.5	19.4 ± 0.2	20.3 ± 1.3	22 ± 2.1	20.1 ± 1.4	20 ± 1.4
LVEDd (mm)	46.4 ± 2.8	42.4 ± 3.7	42.2 ± 1.8	37.6 ± 1.6	46 ± 1.2	40.6 ± 2.3	49.8 ± 2.5	48.6 ± 1.5	51 ± 1.3 ^a^	48 ± 1.8 ^e^	58.5 ± 3.5 ^a^	51.1 ± 2.1 ^h^
LVPW (mm)	21.4 ± 0.8	26.2 ± 2.4 ^c^	20.8 ± 1.2	26.2 ± 2.3 ^f^	20.2 ± 0.5	27 ± 2.7 ^i^	22.1 ± 0.8	29.8 ± 1.0 ^d^	22.5 ± 1.1	28.5 ± 1.7 ^g^	21.6 ± 1	26.1 ± 1.2 ^j^
LVEDs (mm)	22 ± 0.8	19 ± 1.4	19.6 ± 1.4	15.4 ± 1.5	22.6 ± 1.0	16.6 ± 1.9	26.1 ± 2.4	25.1 ± 1.5 ^b^	24.6 ± 1.8	24 ± 2.7 ^e^	34 ± 4 ^a^	28.3 ± 2 ^h^
HR (bpm)	341.6 ± 15.6	276.2 ± 18.52 ^c^	342 ± 53.5	265 ± 29.4	341 ± 19.9	225.2 ± 28.9 ^i^	295.2 ± 5.7 ^a^	245.4 ± 15.9 ^d^	324 ± 27.8	272.9 ± 29.6	314.7 ± 22.9	253.3 ± 21.2
%EF	88.3 ± 1.9	89.3 ± 1.8	89.4 ± 1.5	90.4 ± 2.9	85.8 ± 2.6	85.5 ± 5.9	83.2 ± 4.3	83.6 ± 2.9	87.5 ± 2.5	85.4 ± 3.7	77.3 ± 4.7	79.9 ± 3.9
%FS	52.1 ± 2.4	54.6 ± 2.9	53.3 ± 3.4	58.6 ± 4.6	50.5 ± 3.2	59.5± 2.9	47.4 ± 4.3	48.1 ± 3	51.8 ± 2.7	50.5 ± 4.3	42.4 ± 4.5	44.5 ± 3.8 ^h^

Data are the mean ± SE of 5 different animals in each experimental group. Two-way ANOVA was performed followed Fisher’s LSD test and a *p* ≤ 0.05 was considered statistically significant. ^a^ Baseline Intact vs. Oopho; ^b^ SS Intact vs. Oopho; ^c^ Intact Baseline vs. SS; ^d^ Oopho Baseline vs. SS; ^e^ Met A Intact vs. Oopho; ^f^ Intact Baseline vs. Met A; ^g^ Oopho Baseline vs. Met A; ^h^ Met D Intact vs. Oopho; ^i^ Intact Baseline vs. Met D; ^j^ Oopho Baseline vs. Met D. IVS: Interventricular septum; LVEDd: LV dimensional at end-diastole; LVEDs: LV dimension at end-systole; LVPW: LV dimension posterior wall; EF: Ejection fraction; FS: Fractional shortening: HR: Heart rate; bpm: beats per minute.

**Table 4:** Antioxidant and oxidative stress markers in homogenate total from heart rats exposed to Met A and Met D.

	Intact Rats			Oopho Rats		
Marker	SSI	Met A	Met D	SSI	Met A	Met D
NO (pmoles/mL)	61.5 ± 11.7	81.8 ± 11.5	31.8 ± 14.9 ^c^	34.5 ± 8.4	37.1 ± 11.3 ^b^	34 ± 11.8
TAC (mmol/L)	614.9 ± 158.7	821.3 ± 144.2	307.9 ± 143.3 ^c^	348.7 ± 91.6	522.8 ± 141.6	376 ± 147.3
BH2 (pmoles/mL)	0.44 ± 0.1	0.35 ± 0.06	1.86 ± 0.9 ^c^	0.97 ± 0.3	1.02 ± 0.5 ^b^	1.09 ± 0.3
BH4 (pmoles/mL)	0.47 ± 0.09	0.74 ± 0.13	0.26 ± 0.14 ^c^	0.24 ± 0.05	0.32 ± 0.09 ^b^	0.27 ±0.09
MDA (pmoles/mL)	0.39 ± 0.18	0.55 ± 0.28	0.37 ± 0.24	0.068 ± 0.02	0.7 ± 0.22 ^d^	0.14 ± 0.03
Malonate (pmoles/mL)	0.22 ± 0.08	0.17 ± 0.1	0.6 ± 0.35	0.73 ± 0.19 ^a^	0.07 ± 0.02 ^d^	0.32 ± 0.07
8HO2dG (pM)	0.15 ± 0.09	0.15 ± 0.03	0.13 ± 0.01	0.11 ± 0.02	0.18 ± 0.05	0.22 ± 0.08

Data are the mean ± SE of 5 different animals in each experimental group. Two-way ANOVA was performed followed Fisher’s LSD test and a *p* ≤ 0.05 was considered statistically significant. ^a^ SSI Intact vs. Oopho; ^b^ Met A Intact vs. Oopho; ^c^ Intact Met A vs. Met D; ^d^ Oopho SSI vs. Met A. NO: Nitric oxide; TAC: Total antioxidant capacity; BH2: Dihydrobiopterin; BH4: Tetrahydrobiopterin; MDA: Malondialdehyde; 8HO2dG: 8-hydroxy-2-deoxy-guanosine.

## Data Availability

The raw data are available under request due to privacy.

## Supplementary Materials

The following supporting information can be downloaded at: https://www.mdpi.com/article/10.3390/metabo16090647/s1, Supplementary Figure S1: Representative echoes. Supplementary Figure S2: Representative images indicating the administration sites for EDMs.

## References

[1] Thomas M.P., Potter B.V.L. (2013). The Structural Biology of Oestrogen Metabolism. J. Steroid Biochem. Mol. Biol..

[2] Uribe-Alvarez C., Lira-Silva E., Morales-García L., Chiquete-Felix N., Roldán-Gómez F.J., Vargas-Barrón J., García-Trejo J.J., Silva-Palacios A., Uribe-Carvajal S., Pavón N. (2025). Estrogen Degradation Metabolites: Some Effects on Heart Mitochondria. J. Xenobiotics.

[3] Patel S., Homaei A., Raju A.B., Meher B.R. (2018). Estrogen: The Necessary Evil for Human Health, and Ways to Tame It. Biomed. Pharmacother..

[4] Lira-Silva E., Del Valle Mondragón L., Pérez-Torres I., Posadas-Sánchez R., Gómez F.J., Posadas-Romero C., Vargas-Barrón J., Pavón N. (2023). Possible Implication of Estrogenic Compounds on Heart Disease in Menopausal Women. Biomed. Pharmacother..

[5] Liehr J.G., Ricci M.J., Jefcoate C.R., Hannigan E.V., Hokanson J.A., Zhu B.T. (1995). 4-Hydroxylation of Estradiol by Human Uterine Myometrium and Myoma Microsomes: Implications for the Mechanism of Uterine Tumorigenesis. Proc. Natl. Acad. Sci. USA.

[6] Liehr J.G., Ricci M.J. (1996). 4-Hydroxylation of Estrogens as Marker of Human Mammary Tumors. Proc. Natl. Acad. Sci. USA.

[7] Gaikwad N.W., Yang L., Pruthi S., Ingle J.N., Sandhu N., Rogan E.G., Cavalieri E.L. (2009). Urine Biomarkers of Risk in the Molecular Etiology of Breast Cancer. Breast Cancer Basic Clin. Res..

[8] Muñoz-Castañeda J.R., Túnez I., Muñoz M.C., Bujalance I., Muntané J., Montilla P. (2005). Effect of Catecholestrogen Administration during Adriamycin-Induced Cardiomyopathy in Ovariectomized Rat. Free Radic. Res..

[9] Schneider J., Huh M.M., Bradlow H.L., Fishman J. (1984). Antiestrogen Action of 2-Hydroxyestrone on MCF-7 Human Breast Cancer Cells. J. Biol. Chem..

[10] Xu S., Sun J., Zhang Y., Ji J., Sun X. (2021). Opposite Estrogen Effects of Estrone and 2-Hydroxyestrone on MCF-7 Sensitivity to the Cytotoxic Action of Cell Growth, Oxidative Stress and Inflammation Activity. Ecotoxicol. Environ. Saf..

[11] Kim H., Xue X. (2020). Detection of Total Reactive Oxygen Species in Adherent Cells by 2′,7′-Dichlorodihydrofluorescein Diacetate Staining. J. Vis. Exp..

[12] Cardenas M., Alvarez F., Cabrera-Orefice A., Paredes-Carbajal C., Silva-Palacios A., Uribe-Carvajal S., García-Trejo J.J., Pavón N. (2024). Cross-Sex Hormonal Replacement: Some Effects over Mitochondria. J. Steroid Biochem. Mol. Biol..

[13] Pavón N., Cabrera-Orefice A., Gallardo-Pérez J.C., Uribe-Alvarez C., Rivero-Segura N.A., Vazquez-Martínez E.R., Cerbón M., Martínez-Abundis E., Torres-Narvaez J.C., Martínez-Memije R. (2017). In Female Rat Heart Mitochondria, Oophorectomy Results in Loss of Oxidative Phosphorylation. J. Endocrinol..

[14] Prieto-Carrasco R., Silva-Palacios A., Rojas-Morales P., Aparicio-Trejo O.E., Medina-Reyes E.I., Hernández-Cruz E.Y., Sánchez-Garibay C., Salinas-Lara C., Pavón N., Roldán F.J. (2021). Unilateral Ureteral Obstruction for 28 Days in Rats Is Not Associated with Changes in Cardiac Function or Alterations in Mitochondrial Function. Biology.

[15] Silva-Palacios A., Zúñiga-Muñoz A.M., Soria-Castro E., Álvarez-León E., Nieto M., Navarrete-Anastasio G., Carbó R., García-Niño W.R., López-Cervantes P.S., Salas-Venegas V. (2025). Cardioprotective Effect of Senotherapy in Chronically Obese Middle-Aged Female Rats May Be Mediated by a MERCSs/Nrf2 Interaction. J. Nutr. Biochem..

[16] Apak R., Güçlü K., Özyürek M., Karademi˙r S.E., Altun M. (2005). Total Antioxidant Capacity Assay of Human Serum Using Copper(II)-Neocuproine as Chromogenic Oxidant: The CUPRAC Method. Free Radic. Res..

[17] Claeson K., Åberg F., Karlberg B. (2000). Free Malondialdehyde Determination in Rat Brain Tissue by Capillary Zone Electrophoresis: Evaluation of Two Protein Removal Procedures. J. Chromatogr. B Biomed. Sci. Appl..

[18] Kvasnicová V., Samcová E., Jursová A., Jelínek I. (2003). Determination of 8-Hydroxy-2′-Deoxyguanosine in Untreated Urine by Capillary Electrophoresis with UV Detection. J. Chromatogr. A.

[19] Grohe C., Kahlert S., Löbbert K., Meyer R., Linz K.W., Karas R.H., Vetter H. (1996). Modulation of Hypertensive Heart Disease by Estrogen. Steroids.

[20] Sun X., Hao X., Jia Y.-C., Zhang Q., Zhu Y.-Y., Yang Y.X., Zhu B.T. (2025). Protective Effect of 2-Hydroxyestrone and 2-Hydroxyestradiol against Chemically Induced Hepatotoxicity in Vitro and in Vivo. J. Pharmacol. Exp. Ther..

[21] Yan S., Chen Y., Dong M., Song W., Belcher S.M., Wang H.-S. (2011). Bisphenol A and 17β-Estradiol Promote Arrhythmia in the Female Heart via Alteration of Calcium Handling. PLoS ONE.

[22] Xu W., Billon C., Li H., Wilderman A., Qi L., Graves A., Rideb J.R.D.C., Zhao Y., Hayes M., Yu K. (2024). Novel Pan-ERR Agonists Ameliorate Heart Failure Through Enhancing Cardiac Fatty Acid Metabolism and Mitochondrial Function. Circulation.

[23] Phungphong S., Kijtawornrat A., Wattanapermpool J., Bupha-Intr T. (2020). Improvement in Cardiac Function of Ovariectomized Rats by Antioxidant Tempol. Free Radic. Biol. Med..

[24] Phungphong S., Suthivanich P., Boonhoh W., Punsawad C., Cheng Z., Bupha-Intr T. (2025). Targeting NLRP3 Inflammasome Attenuates Cardiac Pyroptosis and Fibrosis in Estrogen-Deficient Diabetic Rats. Pflug. Arch.-Eur. J. Physiol..

[25] Du J., Liu J., Wang X., Wang X., Ma Y., Zhang S., Li Z., Ma J., Liu J. (2025). The Role of Estrogen in the Sex Difference for the Risk Factors of Heart Failure with Preserved Ejection Fraction. Biol. Direct.

[26] Cheng T.-C., Philip J.L., Tabima D.M., Kumari S., Yakubov B., Frump A.L., Hacker T.A., Bellofiore A., Li R., Sun X. (2020). Estrogen Receptor-α Prevents Right Ventricular Diastolic Dysfunction and Fibrosis in Female Rats. Am. J. Physiol.-Heart Circ. Physiol..

[27] Tong D., Schiattarella G.G., Jiang N., May H.I., Lavandero S., Gillette T.G., Hill J.A. (2019). Female Sex Is Protective in a Preclinical Model of Heart Failure With Preserved Ejection Fraction. Circulation.

[28] Cao Y., Vergnes L., Wang Y.-C., Pan C., Chella Krishnan K., Moore T.M., Rosa-Garrido M., Kimball T.H., Zhou Z., Charugundla S. (2022). Sex Differences in Heart Mitochondria Regulate Diastolic Dysfunction. Nat. Commun..

[29] Hernández I., Delgado J.L., Díaz J., Quesada T., Teruel M.J.G., Llanos M.C., Carbonell L.F. (2000). 17β-Estradiol Prevents Oxidative Stress and Decreases Blood Pressure in Ovariectomized Rats. Am. J. Physiol.-Regul. Integr. Comp. Physiol..

[30] Ngo V., Duennwald M.L. (2022). Nrf2 and Oxidative Stress: A General Overview of Mechanisms and Implications in Human Disease. Antioxidants.

[31] Janaszak-Jasiecka A., Płoska A., Wierońska J.M., Dobrucki L.W., Kalinowski L. (2023). Endothelial Dysfunction Due to eNOS Uncoupling: Molecular Mechanisms as Potential Therapeutic Targets. Cell. Mol. Biol. Lett..

[32] Silva-Palacios A., Ostolga-Chavarría M., Sánchez-Garibay C., Rojas-Morales P., Galván-Arzate S., Buelna-Chontal M., Pavón N., Pedraza-Chaverrí J., Königsberg M., Zazueta C. (2019). Sulforaphane Protects from Myocardial Ischemia-Reperfusion Damage through the Balanced Activation of Nrf2/AhR. Free Radic. Biol. Med..

[33] Jaara H.S., Torres S. (2024). Mitochondrial ROS, a Trigger for Mitochondrial Dysfunction and Inflammasome Activation and a Therapeutic Target in Liver Diseases. Explor. Dig. Dis..

[34] Kampaengsri T., Ponpuak M., Wattanapermpool J., Bupha-Intr T. (2021). Deficit of Female Sex Hormones Desensitizes Rat Cardiac Mitophagy. Chin. J. Physiol..

[35] Zhao W., Hou Y., Song X., Wang L., Zhang F., Zhang H., Yu H., Zhou Y. (2021). Estrogen Deficiency Induces Mitochondrial Damage Prior to Emergence of Cognitive Deficits in a Postmenopausal Mouse Model. Front. Aging Neurosci..

[36] Yan X., Ma D., Li R., Xu J., Zou Y., He M. (2025). The Role of Mitochondrial Dysfunction in Ovarian Granulosa Cells in Polycystic Ovary Syndrome. Endocr. Connect..

[37] Yamada K., Ito M., Nunomura H., Nishigori T., Furuta A., Yoshida M., Yamaki A., Shozu K., Yasuda I., Tsuda S. (2025). Interplay of Oxidative Stress, Autophagy, and Rubicon in Ovarian Follicle Dynamics: Orchestrating Ovarian Aging. Antioxidants.

[38] Shorthill S.K., Jones T.L.M., Woulfe K.C., Cherrington B.D., Bruns D.R. (2024). The Influence of Estrogen on Myocardial Post-Translational Modifications and Cardiac Function in Women. Can. J. Physiol. Pharmacol..

