# Supplementary material for: An Exploratory Study on Two Exogenous Estrogenic Metabolites (EDMs): Their Effects In Vitro and on Oophorectomized Female Rats
Source: Metabolites. 2026 Sep 3;16(9):647. doi: 10.3390/metabo16090647 (PMC13609641; doi:10.3390/metabo16090647)
Supplement: Supplementary file 1 [file metabolites-16-00647-s001.zip › metabolites-4469133-supplementary.pdf]

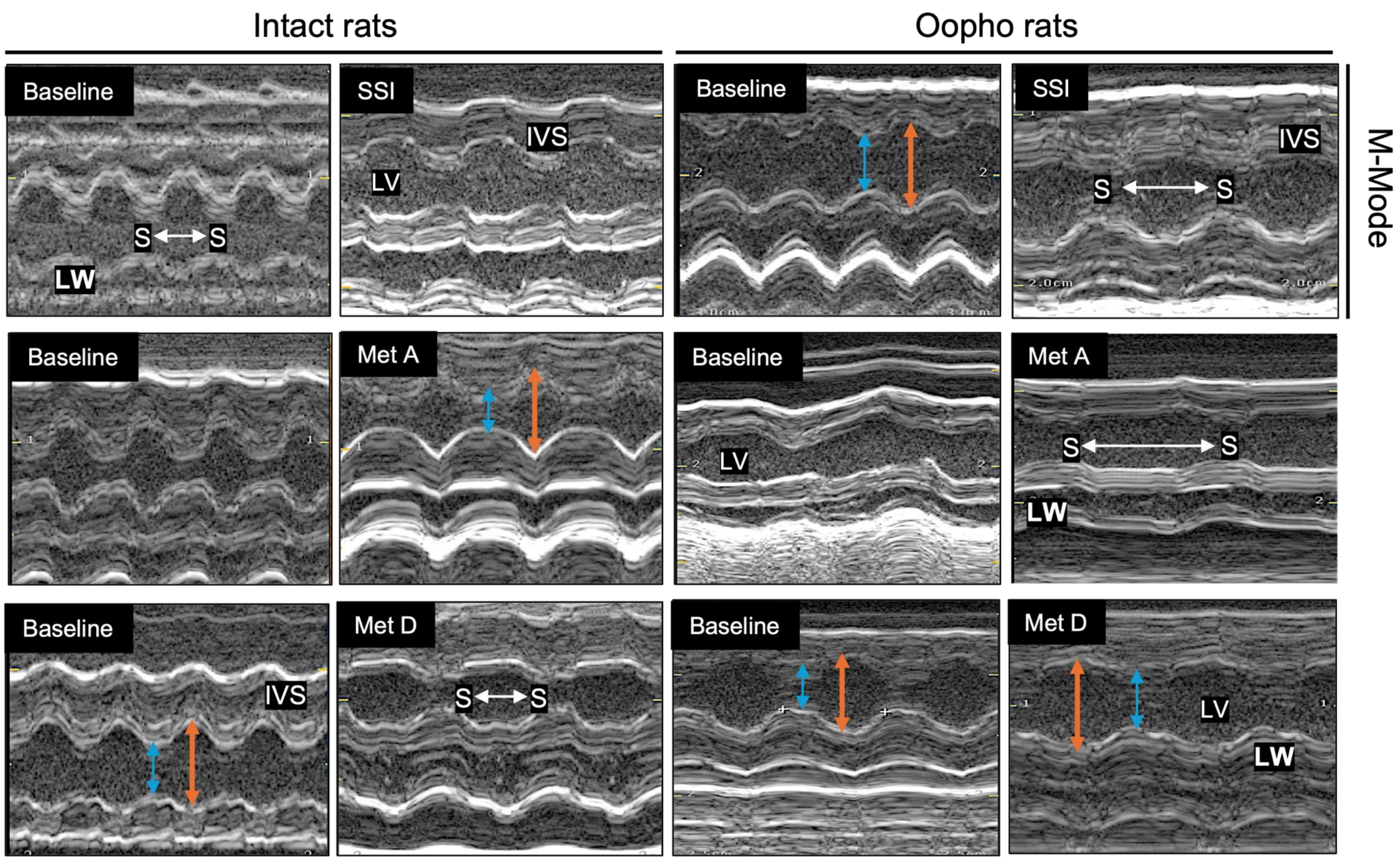

Figure S1. Representative two-dimensional echocardiographic images of the parasternal long-axis view in each group. in M-mode. The end-systolic and end-diastolic diameters of the left ventricle (LV) are show in blue double-headed arrows and orange double-headed arrow, respectively. The thickness of the interventricular septum (IVS), lateral wall (PW), and heart rate (meaured by the distance between two consecutive systoles, S, white double-headed arrow) are also plotted.

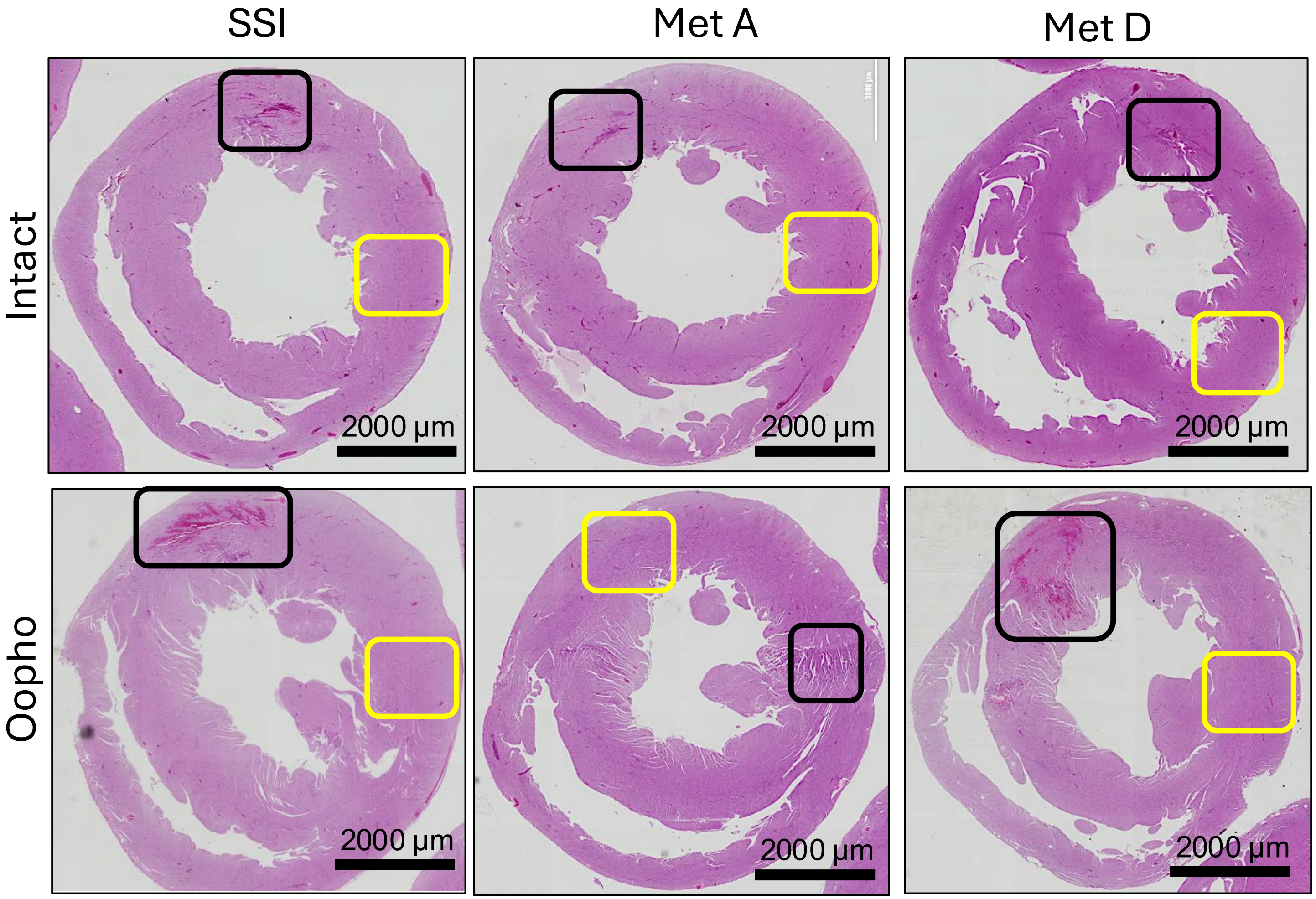

Figure S2. The black boxes indicate the site of administration of the SSI and the metabolites, while the boxes indicate the approximate area of analysis for H&E and Masson's trichrome staining.
